# Urease is an essential component of the acid response network of *Staphylococcus aureus* and is required for a persistent murine kidney infection

**DOI:** 10.1371/journal.ppat.1007538

**Published:** 2019-01-04

**Authors:** Chunyi Zhou, Fatema Bhinderwala, McKenzie K. Lehman, Vinai C. Thomas, Sujata S. Chaudhari, Kelsey J. Yamada, Kirk W. Foster, Robert Powers, Tammy Kielian, Paul D. Fey

**Affiliations:** 1 Department of Pathology and Microbiology, University of Nebraska Medical Center, Omaha, Nebraska, United States of America; 2 Department of Chemistry, University of Nebraska-Lincoln, Lincoln, Nebraska, United States of America; 3 Nebraska Center for Integrated Biomolecular Communication, University of Nebraska-Lincoln, Lincoln, Nebraska, United States of America; University of Tubingen, GERMANY

## Abstract

*Staphylococcus aureus* causes acute and chronic infections resulting in significant morbidity. Urease, an enzyme that generates NH_3_ and CO_2_ from urea, is key to pH homeostasis in bacterial pathogens under acidic stress and nitrogen limitation. However, the function of urease in *S*. *aureus* niche colonization and nitrogen metabolism has not been extensively studied. We discovered that urease is essential for pH homeostasis and viability in urea-rich environments under weak acid stress. The regulation of urease transcription by CcpA, Agr, and CodY was identified in this study, implying a complex network that controls urease expression in response to changes in metabolic flux. In addition, it was determined that the endogenous urea derived from arginine is not a significant contributor to the intracellular nitrogen pool in non-acidic conditions. Furthermore, we found that during a murine chronic renal infection, urease facilitates *S*. *aureus* persistence by promoting bacterial fitness in the low-pH, urea-rich kidney. Overall, our study establishes that urease in *S*. *aureus* is not only a primary component of the acid response network but also an important factor required for persistent murine renal infections.

## Author summary

Urease has been reported to be crucial to bacteria in environmental adaptation, virulence, and defense against host immunity. Although the function of urease in *S*. *aureus* is not clear, recent evidence suggests that urease is important for acid resistance in various niches. Our study deciphered a function of *S*. *aureus* urease both in laboratory conditions and during host colonization. Furthermore, we uncovered the major components of the regulatory system that fine-tunes the expression of urease. Collectively, this study established the dual function of urease which serves as a significant part of the *S*. *aureus* acid response while also serving as an enzyme required for persistent kidney infections and potential subsequent staphylococcal metastasis.

## Introduction

Bacterial pathogens often encounter acidic environments within host tissues and employ several direct and indirect defense measures [1]. Direct measures include the utilization of proton pumps and generation of alkaline compounds such as ammonia to neutralize pH. Indirect methods such as damage repair, biofilm formation, and metabolic alterations, are utilized to rescue cell viability. *Staphylococcus aureus* is a leading cause of opportunistic infections in community and health care settings [2, 3]. *S*. *aureus* resides in multiple acidic niches during colonization and infection of the human host that include the surface of the skin and within abscesses [4–6]. It is important to note that *S*. *aureus* is sensitive to acetic acid stress when growing in the presence of excess glucose [7]. Weak acids such as acetic acid are unique in potentiating stationary phase cell death, in that unlike strong acids that fully dissociate in water, the undissociated weak acids can easily enter into the cytoplasm and reduce the intracellular pH by releasing protons. Therefore, *S*. *aureus* must overcome different kinds of acid stress to maintain viability. However, mechanisms of acid resistance in *S*. *aureus* are not well described. It has been shown that *sodA*, which encodes a superoxide dismutase, is induced upon acid stress and facilitates acid tolerance by alleviating the cell damage caused by reactive oxygen species [8]. In addition, a σ^B^–dependent acid-adaptive response has been described that facilitates *S*. *aureus* survival in media with a pH of 2 after pre-exposure to a sub-lethal pH of 4 [9]. Based on global transcriptional studies, increased urease activity is thought to be a major contributor to acid resistance in *S*. *aureus* [10–13].

In humans, urea is produced in the liver via the urea cycle as a means to remove excess nitrogen. Urea enters the bloodstream, becomes concentrated in the kidneys, and is excreted during urination. The concentration of urea in the blood is normally 2.5–7.1 mM, and it is found in other body fluids such as gastric acid, sweat, and saliva. The level of urea in the saliva is 3–10 mM in healthy individuals but can reach 15 mM in patients with renal diseases [14]. Notably, the re-absorbance of urea from the collecting ducts makes the interstitium of the kidney inner medulla a urea-rich environment.

Urease (EC: 3.5.1.5) is a nickel-dependent metalloenzyme that catalyzes the hydrolysis of urea into ammonia (NH_3_) and carbon dioxide (CO_2_) [15–17]. For some bacterial species, urease is an integral part of the bacterial acid response network, as the hydrolysis product ammonia is readily protonated into ammonium (NH_4_^+^), during which process protons are consumed, resulting in an increase in pH [1]. Urease is crucial for niche adaptation of many bacterial pathogens. For example, urease is essential for the survival of *Helicobacter pylori* in the stomach lining, where the pH can be as low as 2.5 [18]. With a high affinity for urea, urease from *H*. *pylori* is required not only for the establishment of infections but also for the maintenance of a chronic infection [19]. Also, *Streptococcus salivarius* produces urease to utilize salivary urea as a nitrogen source for growth while resisting acid stress [20]. Over 90% of *S*. *aureus* strains are urease-producing [21], which is encoded by the urease gene cluster *ureABCEFGD*. The α, β, and γ subunits that comprise the apoenzyme are encoded by *ureC*, *ureB*, and *ureA*, whereas *ureEFGD* genes encode accessory proteins. Previous studies have shown that urease genes are highly transcribed during biofilm growth conditions [13, 22]. However, the function and utilization of urease in *S*. *aureus* has not been comprehensively studied.

In this work, we explored the *in vitro* and *in vivo* functions of urease in *S*. *aureus*. We found that *S*. *aureus* primarily utilizes urease to facilitate pH homeostasis under weak acidic stress, but it does not utilize urea as a nitrogen source under neutral pH. Lastly, our data demonstrate that urease is essential for *S*. *aureus* to persist in mouse kidneys, where a significant pH gradient exists and urea is an abundant nitrogen source.

## Results

### Urease rescues cell death potentiated by acetic acid via ammonia generation in the presence of exogenous urea

**Fig 1:**
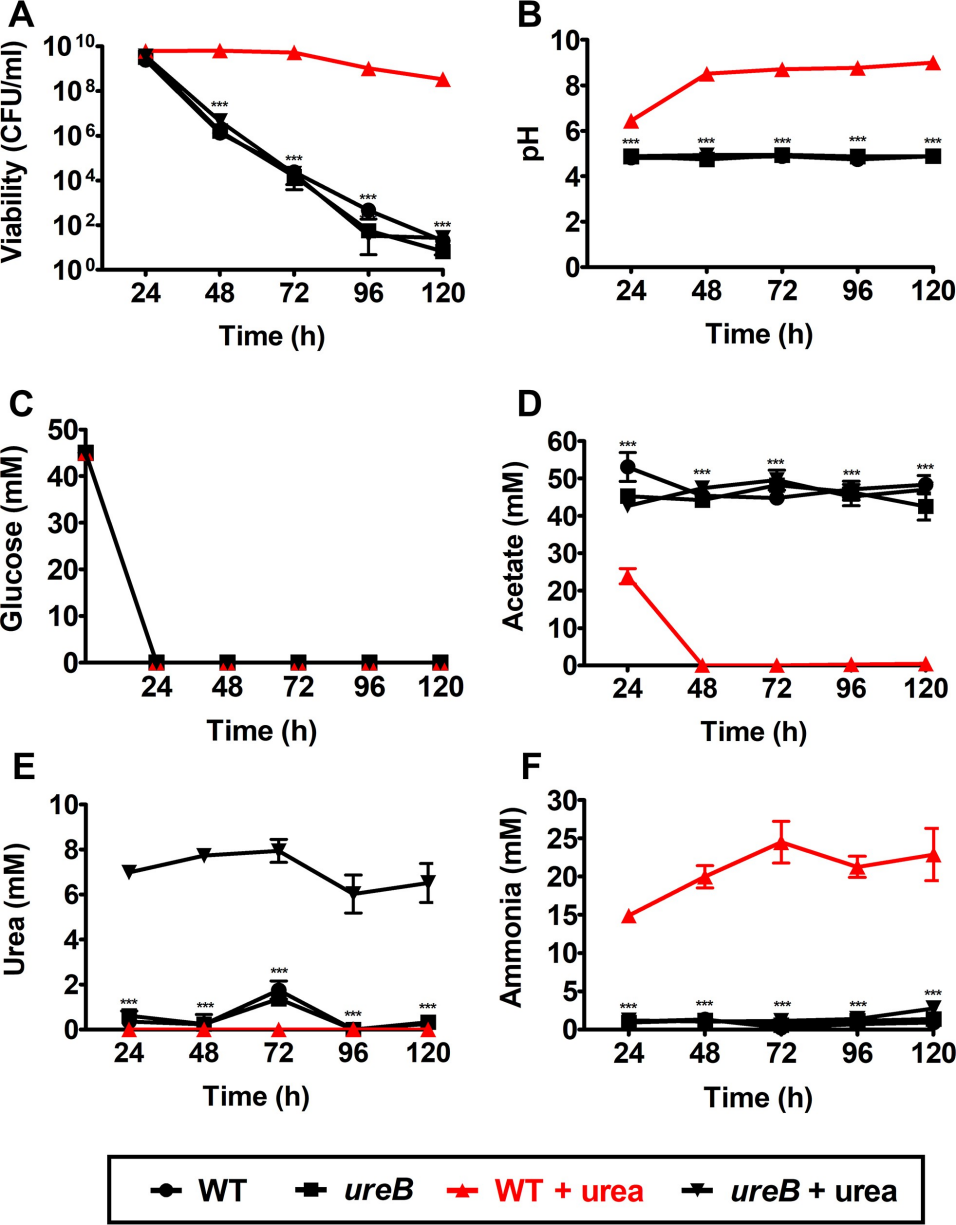
Urease rescues cell death potentiated by acetic acid via ammonia generation in the presence of exogenous urea. Three biological replicates of *S*. *aureus* JE2 WT and JE2 *ureB*::*ΦΝΣ* were cultured in TSB containing 45 mM glucose with and without 10 mM urea over 5 days. Every 24 h, (**A**) cell viability (CFU/ml), (**B**) extracellular pH, (**C**) extracellular glucose levels (mM), (**D**) extracellular acetate levels (mM), (**E**) extracellular urea levels (mM), and (**F**) extracellular ammonia levels (mM) were monitored and plotted with mean ± standard error of the mean [SEM]). Statistical significance was assessed using two-way repeated measures ANOVA, followed by Bonferroni post-test compared to WT + urea in **(A), (B), (D), (F),** and to *ureB +* urea in **(E)** at each timepoint; *** *P <* 0.001.

**Fig 2:**
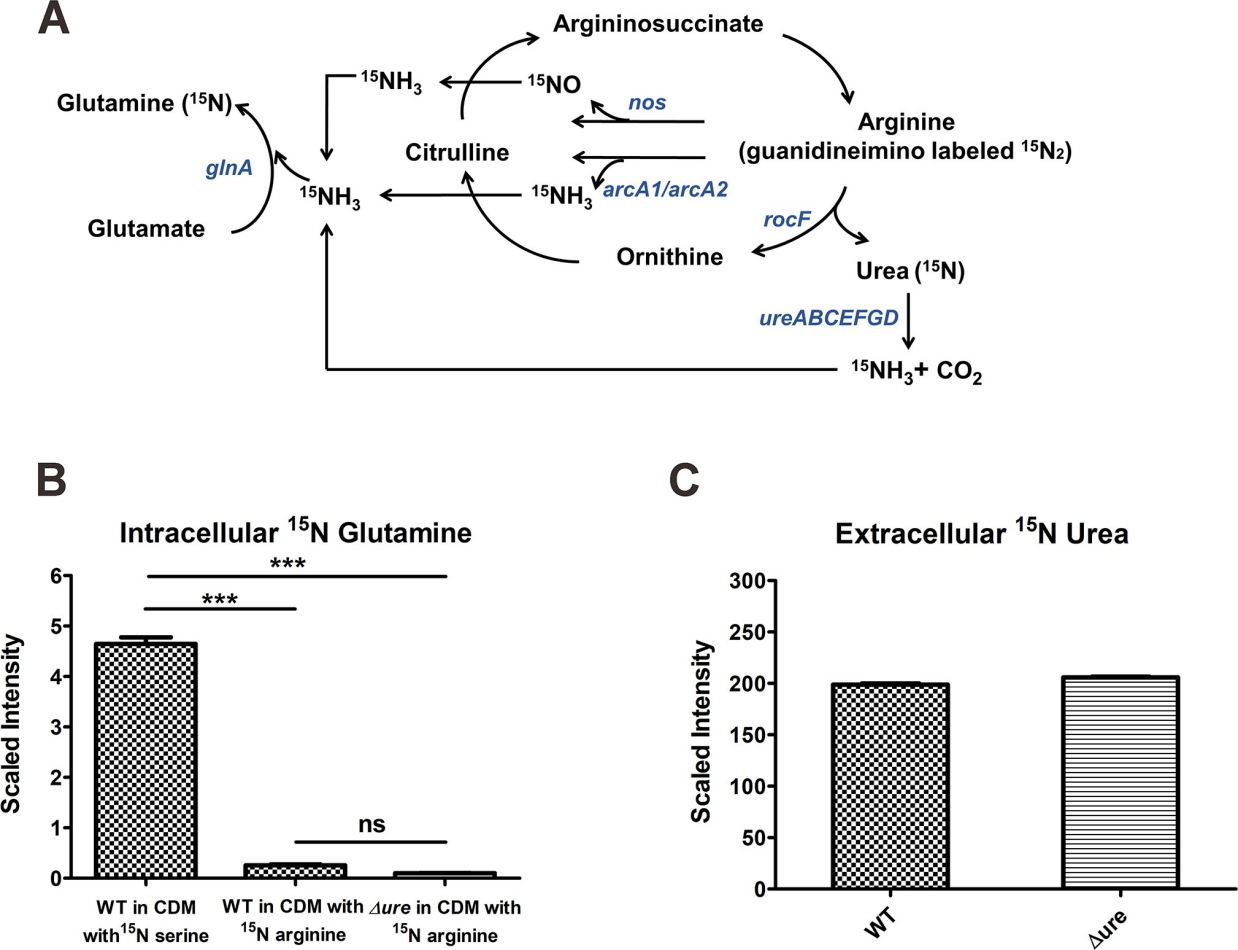
Endogenous urea is not utilized as a nitrogen source. (**A**) *S*. *aureus* arginine catabolism and nitrogen flow (*nos* encodes nitric oxide synthase, *arcA1/arcA2* encodes arginine deiminases, *rocF* encodes arginase, *ureABCEFGD* encodes urease, and *glnA* encodes glutamine synthetase). (**B**) JE2 WT was cultured in CDM containing ^15^N-serine whereas both JE2 WT and JE2 *Δure* were cultured in CDM containing ^15^N-arginine. The intracellular ^15^N-glutamine levels were measured by NMR (n = 5/strain, mean ± SEM). Statistical significance was assessed using one-way ANOVA followed by Tukey's post-test; *** *P <* 0.001; ns, not significant. (**C**) WT and *Δure* were cultured in CDM with ^15^N-arginine, and the extracellular ^15^N-urea levels were measured by NMR (n = 5/strain, mean ± SEM).

Previous work has documented that aerobic growth of *S*. *aureus* in tryptic soy broth (TSB) containing excess glucose (35–45 mM) impairs stationary phase survival of *S*. *aureus* [7]. Under these growth conditions, the acetate derived from glucose catabolism is not consumed as a secondary carbon source, and the pH in the medium remains low, which potentiates cell death. Based on those observations, we hypothesized that the presence of urea in TSB containing 45 mM glucose would rescue cell death due to ammonia generation via urease activity. To test this hypothesis, we performed a growth assay in which JE2 wildtype (WT) and JE2 *ureB*::*ΦΝΣ* (*ureB*) were aerobically cultured in TSB containing 45 mM glucose with or without 10 mM urea. Over the period of 120 h, colony forming units (CFU/ml) and extracellular pH were monitored every 24 h. In addition, culture supernatant was analyzed to measure glucose, acetate, urea, and ammonia concentrations. In the absence of urea supplementation, both WT and the *ureB* mutant showed a drastic decrease in cell viability (~9 log_10_ difference) while maintaining an acidic extracellular pH (~4.8) (Fig 1A and 1B). However, we observed a urease-dependent increase in viability (~8 log_10_ difference) and medium pH (~4 pH unit difference) in the presence of exogenous urea (Fig 1A and 1B). Both pH and viability phenotypes of the *ureB* mutant were complementable by integrating *ureABCEFGD* into the chromosomal SaPI1 *attC* site (S1 Fig). Glucose in the medium was depleted by 24 h for both WT and the *ureB* mutant either with or without urea supplementation (Fig 1C). However, only WT grown in TSB supplemented with 10 mM urea consumed acetate (Fig 1D). Lastly, we observed a urease-dependent consumption of urea coincident with the generation of ~20 mM NH_3_ (Fig 1E and 1F). Thus, these results suggested that the *S*. *aureus* urease functions as part of an acid response network to facilitate pH homeostasis in the presence of urea. Weak acids and subsequent intracellular acidification has been previously shown to generate endogenous reactive oxygen species and potentiate cell death [7]. Thus, to evaluate the physiological status of WT and the *ureB* mutant in the above growth assay, flow cytometry was performed to assess cellular respiration and reactive oxygen species (ROS) levels. The results confirmed that urease-mediated pH homeostasis rescued cellular respiration and protects cells from endogenous ROS under weak acid stress in the presence of urea at 72 h of growth when viability differences are evident (Fig 1A and S2 Fig). It is important to note that in TSB the concentration of arginine, which can be catabolized to generate NH_3_ via arginase/urease, nitric oxide synthase (NOS), or two separate arginine deiminase (ADI) systems (Fig 2A), is not sufficient to rescue the survival of JE2 in this assay. Therefore, we repeated the assay with excess arginine (5 mM) in TSB containing 45 mM glucose. As a result, excess arginine was unable to rescue viability of JE2 to the same extent as urea, although we did observe an arginine deiminase-dependent increase in viability (~2 log_10_) (S3A and S3B Fig). Collectively, these data suggest that urea derived from arginine via RocF and subsequent urease activity is not functional in this assay. However, *Staphylococcus epidermidis* catabolized either arginine (S3C and S3D Fig) [23] or urea (S3E and S3F Fig) to rescue growth under weak acid stress.

### Urease transcription is induced by weak acid stress and regulated by CcpA, Agr, and CodY

**Fig 3:**
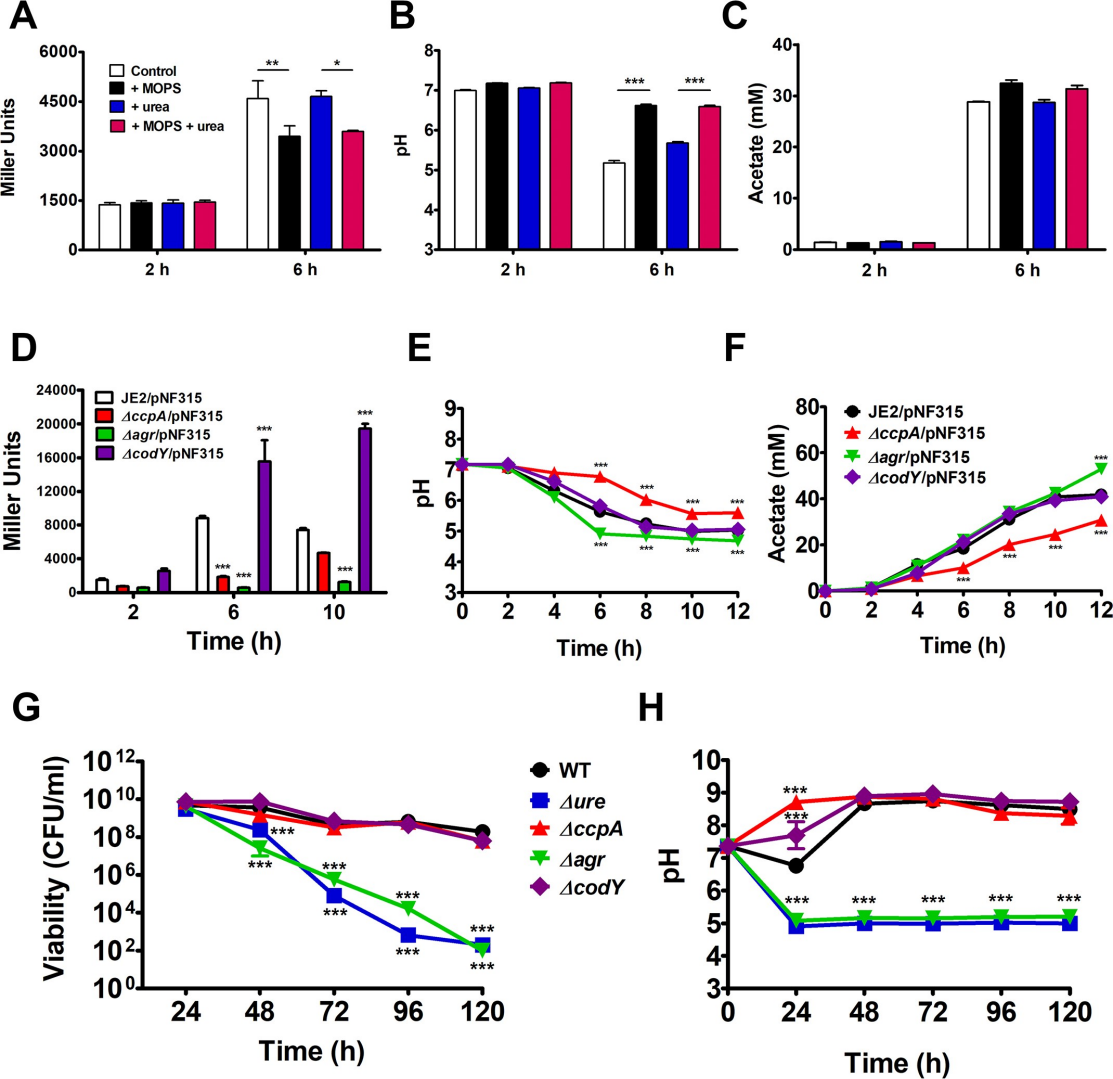
Urease transcription is induced by weak acid stress and regulated by CcpA, Agr, and CodY. (**A**)-(**C**) JE2/pNF315 was cultured in TSB containing 45 mM glucose alone (control), buffered with 100 mM MOPS (+ MOPS), supplemented with 10 mM urea (+ urea), or both (+ MOPS + urea). (**A**) β-galactosidase assays of samples collected at 2 and 6 h. Miller units were normalized with the protein concentrations (n =  3/strain, mean ± SEM). (**B**) pH and (**C**) extracellular acetate concentrations were measured at 2 and 6 h (n =  3/strain, mean ± SEM). Statistical significance was assessed using two-way repeated measures ANOVA; * *P* < 0.05, ** *P <* 0.01, *** *P <* 0.001. (**D**)-(**F**) JE2/pNF315, JE2 *ΔccpA*/pNF315, JE2 *Δagr*/pNF315, and JE2 *ΔcodY*/pNF315 were cultured in TSB containing 45 mM glucose and 10 mM urea. (**D**) β-galactosidase assays of cells collected at 2, 6, and 10 h. Miller units were normalized with the protein concentrations (n = 3/strain, mean ± SEM). (**E**) pH and (**F**) extracellular acetate concentrations were measured at 0–12 h (n =  3/strain, mean ± SEM). Statistical significance was assessed using two-way ANOVA followed by Bonferroni post-test compared to JE2/pNF315 at each timepoint; *** *P <* 0.001. (**G**) and (**H**) Growth assay of JE2 WT, JE2 *Δure*, JE2 *ΔccpA*, JE2 *Δagr*, and JE2 *ΔcodY* in TSB containing 45 mM glucose and 10 mM urea. (**G**) Viability (CFU/ml) and (**H**) pH was monitored every 24 h (n = 3/strain, mean ± SEM). Statistical significance was assessed using two-way ANOVA followed by Bonferroni post-test compared to JE2 WT at each timepoint; *** *P <* 0.001.

To further investigate the transcriptional regulation of the *ure* operon, a *lacZ* reporter plasmid pNF315 was generated in which the promoter of *ure* was fused to the promoterless *lacZ* gene and transduced into JE2. The previous experiments (Fig 1) suggested that *ure* transcription or urease function is induced under weak acid stress [10]. Indeed, as the pH deceased due to the accumulation of acetate, *ure* transcription was induced 3.3-fold at 6 h comparing to 2 h, in TSB containing 45 mM glucose with or without 10 mM urea (Fig 3A–3C). When the media were buffered to a pH of 7.25 with 100 mM 3-(N-morpholino) propanesulfonic acid (MOPS), the transcription of *ure* was significantly inhibited regardless of urea supplementation (Fig 3A). These results indicate that the transcription of urease genes is induced by weak acid stress. Multiple global transcriptional studies have suggested that the accessory gene regulator (Agr) quorum sensing system, as well as global regulators CcpA and CodY, function to regulate the transcription of the urease operon [24–26]. To assess these relationships in our model, JE2 WT, *ΔccpA*::*tetL* (*ΔccpA*), *Δagr*::*tetM* (*Δagr*), and *ΔcodY*::*ermB* (*ΔcodY*) each containing pNF315 were grown aerobically in TSB containing 45 mM glucose and 10 mM urea. β-galactosidase activity assays were performed with cell lysate collected during early- (2 h), mid- (6 h), and post- (10 h) exponential phases of growth. The transcription of the *ure* operon was significantly decreased in *ΔccpA*/pNF315 (6 h) and *Δagr*/pNF315 (6 and 10 h), and significantly increased in *ΔcodY*/pNF315 at 6 and 10 h (Fig 3D), indicating that the transcription of *ure* genes is activated by CcpA and Agr and negatively regulated by CodY. However, it is unclear if *ure* transcriptional regulation by CcpA, Agr, or CodY is via direct or indirect regulation. To corroborate the effects of these regulators on urease activity, JE2 WT, *Δure*, *ΔccpA*, *Δagr* and *ΔcodY* were grown in TSB containing 45 mM glucose and 10 mM urea for 120 h (Fig 3E–3H). As expected based on the transcriptional analysis, *ΔcodY* essentially phenocopied WT with regards to pH, acetate production and viability (Fig 3E–3H). However, since *Δagr* displayed reduced *ure* transcription, it was predicted that the viability would be significantly reduced in the 120-h growth assay. Indeed, the extracellular pH of *Δagr* was significantly different from WT by 6 h of growth (Fig 3E) and remained at 5.1 from 24–120 h similar to *Δure*. Further, in comparison to JE2 WT, the viability of *Δagr* decreased ~6 log_10_ to ~10^2^ CFU/ml at 120 h similar to *Δure*. Lastly, based on Fig 3D and the β-galactosidase activity assay documenting a decrease in *ure* transcription, we would expect the *ΔccpA* mutant to have decreased viability similar to *Δagr* and *Δure*. However, we found that the pH was significantly higher than WT/ *ΔcodY* from 6–12 h of growth (Fig 3E). In addition, it produced less extracellular acetate than WT (Fig 3F), presumably due to consumption of acetyl-CoA via the tricarboxylic acid (TCA) cycle as CcpA represses TCA cycle activity [27–29]. Further, it survived as well as WT, and the pH remained alkaline over the entire 120 h (Fig 3G and 3H).

### Loss of *ccpA* promotes survival under weak acid stress

**Fig 4:**
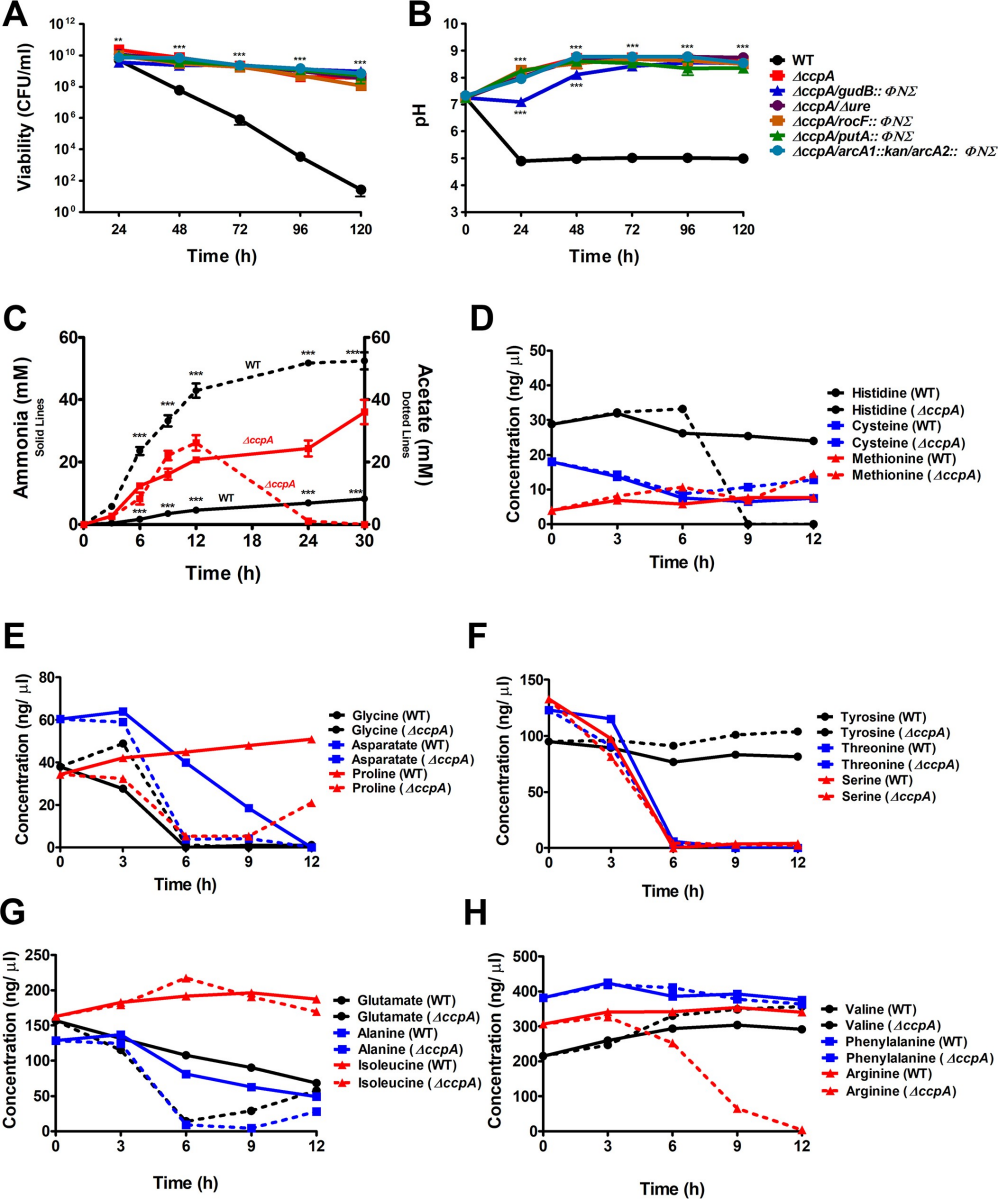
Loss of *ccpA* promotes survival under weak acid stress. (**A**) and (**B**) Five-day growth assay of JE2 WT, JE2 *ΔccpA*, JE2 *ΔccpA/gudB*::*ΦΝΣ*, JE2 *ΔccpA/Δure*, JE2 *ΔccpA/rocF*::*ΦΝΣ*, JE2 *ΔccpA/putA*::*ΦΝΣ*, and JE2 *ΔccpA/arcA1*::*kan/arcA2*::*ΦΝΣ*, cultured in TSB containing 45 mM glucose. (**A**) Viability (CFU/ml) and (**B**) pH was monitored every 24 h (n = 3/strain, mean ± SEM). Statistical significance was assessed using two-way repeated measures ANOVA followed by Bonferroni post-test compared to JE2 WT at each timepoint; ** *P <* 0.01, *** *P <* 0.001. (**C**)-(**H**) Growth assay of JE2 WT and JE2 *ΔccpA* cultured in TSB containing 45 mM glucose over 30 h. (**C**) Extracellular ammonia and acetate levels were measured at 0, 3, 6, 9, 12, 24, and 30 h (n = 3/strain, mean ± SEM). Statistical significance was assessed using two-way ANOVA, followed by Bonferroni post-test; *** *P <* 0.001. (**D**)-(**H**) Amino acid analysis was performed with culture supernatants of both strains at 0, 3, 6, 9, and 12 h to determine the free amino acid concentrations (ng/μl). Amino acid analysis experiments performed with one replicate. WT is represented by solid lines and the *ccpA* mutant is represented by dotted lines.

Based on our previous work documenting the function of CcpA in repressing amino acid catabolism [30], we hypothesized that the survival of the *ΔccpA* mutant in the above growth assay was urease-independent due to derepression of amino acid catabolism and subsequent generation of ammonia. Previous investigations have documented that in the presence of a preferred carbon source such as glucose, CcpA represses amino acid catabolic genes including *gudB* (encoding glutamate dehydrogenase), *rocF* (encoding arginase), *putA* (encoding proline dehydrogenase), and *arcA1/arcA2* (encoding arginine deiminases) [23, 24, 30–32], all of which produce ammonia as a byproduct. To determine whether the survival of *ΔccpA* was dependent upon urease or catabolism of a particular amino acid, a growth assay was performed in which JE2 WT, *ΔccpA*, *ΔccpA/gudB*::*ΦΝΣ*, *ΔccpA/Δure*, *ΔccpA/putA*::*ΦΝΣ*, and *ΔccpA/arcA1*::*kan/arcA2*::*ΦΝΣ* were cultured in TSB containing 45 mM glucose. In the absence of urea, JE2 WT was unable to survive as expected, presumably due to a dramatic decrease in pH observed over the 120 h experimental timeframe (Fig 4A and 4B). However, *ΔccpA/gudB*::*ΦΝΣ*, *ΔccpA/Δure*, *ΔccpA/putA*::*ΦΝΣ*, *ΔccpA/arcA1*::*kan/arcA2*::*ΦΝΣ* all phenocopied *ΔccpA*, suggesting that it was not the catabolism of one specific amino acid that was responsible for cell survival in this assay (Fig 4A and 4B). These data led to the hypothesis that the derepression of overall amino acid catabolism provides the *ΔccpA* mutant a growth advantage. Further, as shown in Fig 3F and previously it is known that *ΔccpA* mutants produce less extracellular acetate due to derepression of the TCA cycle [27–29]. To address this hypothesis, JE2 WT and *ΔccpA* were grown in TSB containing 45 mM glucose. As expected, the *ΔccpA* mutant generated significantly more ammonia and the extracellular acetate was eventually consumed by *ΔccpA* compared to JE2 WT (Fig 4C). Amino acid analysis of the same supernatant demonstrated that the *ΔccpA* mutant consumed histidine, aspartate, proline, glutamate, alanine, and arginine at a faster rate than WT (Fig 4D–4H). Taken together, these data suggest that the absence of CcpA in *S*. *aureus* facilitates survival in the presence of excess glucose due to decreased acetate generation and increased ammonia generation by amino acid catabolism of multiple amino acids.

### Endogenous urea is not utilized as a nitrogen source under non-acidic conditions

In previous experiments using TSB containing 45mM glucose, catabolism of arginine in the media is repressed by CcpA (Fig 4H). Therefore, we were unable to determine the potential function, if any, of endogenously derived urea. Further, it is unclear if urease is active at neutral pH and generates NH_3_ for use in nitrogen metabolism. To determine if urease is functional in media where arginine is rapidly catabolized thus generating urea from arginine via arginase (RocF), we grew JE2 in buffered chemically defined medium (CDM) lacking glucose [32, 33]. CDM is a defined medium that lacks glucose but contains 18 amino acids except glutamine and asparagine [34], buffered at pH 7.5. We reasoned that if urease catalyzes the reaction generating NH_3_ from urea, the ammonia would be actively utilized by glutamine synthetase to synthesize glutamine from glutamate (Fig 2A). Both glutamine and glutamate are major amino donors for cellular reactions [35]. Therefore, JE2 WT and the *ureABCEFGD* deletion mutant (*Δure*) were grown aerobically in CDM containing 0.1 g/L ^15^N-arginine (guanidino-labeled only) and cells were harvested at 7 h, at which time both supernatant and intracellular components were assessed by nuclear magnetic resonance (NMR) to detect ^15^N-glutamine [36]. Since the ammonia generated from amino acid catabolism is also utilized for glutamine synthetase, the rapidly catabolized serine was labeled with ^15^N as a control [31]. As a result, 17 times more ^15^N-glutamine was detected when ^15^N-serine was added to CDM than when ^15^N-arginine was added (Fig 2B). In addition, no difference was noted when comparing the ^15^N-glutamine detected from WT or *Δure* when grown in CDM containing ^15^N-arginine. Therefore the small amount of detected ^15^N-glutamine was derived from ADI or NOS-generated ^15^NH_3_ (Fig 2B). However, in CDM containing ^15^N-labeled arginine, significant ^15^N-labeled urea was detected extracellularly in both WT and *Δure* (Fig 2C), indicating that the nitrogen from arginine catabolism does not enter the intracellular nitrogen pool, but is rather excreted as urea under neutral pH.

### Urease is essential for the persistence of *S*. *aureus* during a mouse chronic renal infection

**Fig 5:**
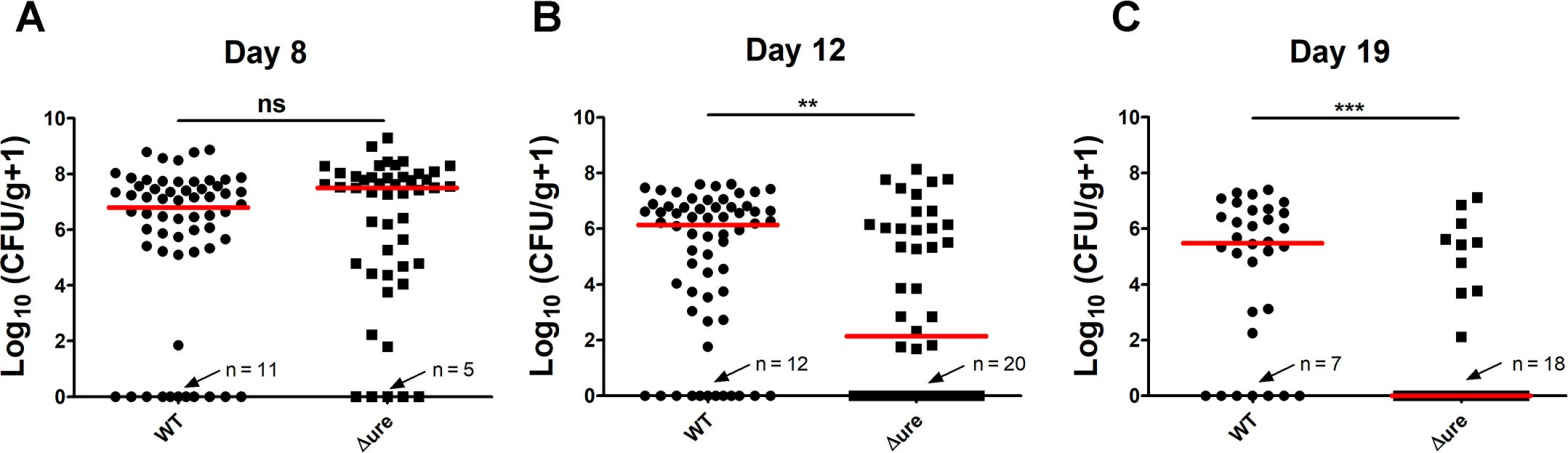
Urease is essential for the persistence of *S*. *aureus* during a murine chronic renal infection. (**A**)-(**C**) *S*. *aureus* murine bacterial model, male and female C57BL/6 mice were infected with JE2 WT and JE2 *Δure*. (**A**) On day 8 (number of mice: WT, n = 30; *Δure*, n = 24), (**B**) day 12 (number of mice: WT, n = 31; *Δure*, n = 23), (**C**) day 19 (number of mice: WT, n = 16; *Δure*, n = 14) post-inoculation, bacterial burdens of were calculated as Log_10_ (CFU/g of tissue +1) and plotted with medians. Statistical significance was assessed using Mann- Whitney test; ** *P <* 0.01, *** *P <* 0.001; ns, not significant. Note that 31 out of 204 mice total died due to the infection but were not further assessed in the analyses.

Our data demonstrated that urease facilitates pH homeostasis and cell survival *in vitro* under weak acid stress in the presence of urea. However, it is unclear if urease functions to facilitate staphylococcal colonization or virulence. One niche where urease may be important is the host skin, where *S*. *aureus* resides within hair follicles and sweat glands [37]. Moreover, it is known that human sweat contains 22.2 mM urea [38] and the pH of human skin is ~4 to ~6 [39]. However, animal models of *S*. *aureus* skin colonization are difficult to replicate since mice and other rodents do not sweat. Therefore, we reasoned that another host niche where urease might be required was in the colonization of the kidney, which has a low tissue pH and a relatively high concentration of urea. In addition, it is well known that *S*. *aureus* causes chronic kidney infections in mice and thus the kidney provides a nidus for subsequent staphylococcal metastasis (S4 Fig) [40]. To test this hypothesis, we used a mouse bacteremia model in which C57BL/6 mice were retro-orbitally injected with JE2 WT and *Δure*. On days 8, 12 and 19 post-infection, bacterial burden in the kidney was determined (Fig 5). Although no difference between WT and *Δure* was noted on day 8 (Fig 5A), kidneys infected with *Δure* had significantly lower bacterial burden on days 12 and 19, with more kidneys below the limit of detection infection compared to WT (Fig 5B and 5C), indicating that urease contributes to the persistence of *S*. *aureus* during a mouse chronic kidney infection. To determine if the host immune response differed between mice infected with either WT or *Δure*, leukocyte populations were assessed from infected kidneys on day 8, an interval where bacterial burdens were equivalent, to prevent bias from animals that had cleared the infection. However, no significant differences were noted between these two groups (S5 Fig) suggesting the absence of *ure* did not skew the immune response to facilitate enhanced clearance.

## Discussion

Acid stress, along with other environmental risk factors such as extreme temperatures, osmotic pressure, and nutrient depletion, is challenging for bacterial survival [41]. Accordingly, a variety of strategies are utilized to resist low pH which also contribute to bacterial virulence [1]. In *Escherichia coli*, four main acid resistance systems (ARs) have been described: the oxidative system AR1, the glutamate-dependent AR2, the arginine-dependent AR3, and the lysine-dependent AR4 [42]. The amino acid-dependent ARs are composed of a decarboxylase which consumes protons, and an inner membrane antiporter which imports the decarboxylase substrate while exporting the product. *Bacillus cereus* activates not only the general stress response genes via σ^B^ but also proton transporters and amino acid decarboxylases, as well as the ADI system that produces ammonia [43]. *H*. *pylori* is known for the ability to proliferate in extremely low pH environments such as the host gastric acid [44, 45]. In addition to amino acid catabolism, enhanced urease activity sustains favorable intracellular pH by generating ammonia [18, 45–48]. *Mycobacterium tuberculosis* resists acid stress through nitrogen assimilation from asparagine hydrolysis [49], as well as urea hydrolysis [50]. However, there is little known about acid resistance in *S*. *aureus* although it proliferates in multiple mildly acidic niches of the human host.

In the aerobic growth assay of *S*. *aureus* cultured in TSB containing 45 mM glucose, the acetate produced via glucose catabolism is excreted into the growth medium (Fig 1D). When the extracellular pH of the growth medium reaches the pKa of acetic acid (4.8), acetate becomes protonated and is able to traverse the cell membrane and release the proton in the near neutral pH of the cytoplasm. The drop in intracellular pH potentiates cell death in *S*. *aureus* by intracellular acidification and ROS generation [7]. We documented that in the presence of urea, urease functions to facilitate pH homeostasis in a weak acid environment through the generation of ammonia that inhibits the acetate-dependent intracellular acidification (Fig 1E and 1F) [7]. In addition, it was confirmed that the urease activity and subsequent NH_3_ generation rescued cellular respiration and prevented endogenous ROS generation (S2 Fig). The ammonia generated facilitated acetate consumption through acetyl-CoA synthetase and the TCA cycle for subsequent cell growth. Collectively, these data suggest that urease is a significant component of the acid response network of *S*. *aureus* in the presence of urea.

Many gram-positive species, including *S*. *epidermidis*, utilize arginine catabolism as a rapid mechanism to generate ammonia during acidic pH stress [23, 51]. The pathway most utilized is the ADI pathway yielding ammonia, ornithine, and ATP. However, in contrast to *S*. *epidermidis* [23] (S3C and S3D Fig), excess arginine was unable to remarkably rescue *S*. *aureus* during weak acid stress suggesting that ammonia generating pathways via arginine are not significantly active in our aerobic assay containing glucose (S3A and S3B Fig). These results agree with a previous report documenting that the ADI pathway genes are not significantly induced under weak acid stress in *S*. *aureus* [10]. In this work we also confirmed that in *S*. *epidermidis* 1457, additional arginine and urea provided a growth advantage under weak acid stress (S3C and S3E Fig), suggesting that both ADI and urease are active in *S*. *epidermidis*. This result is consistent with another study documenting the differential transcriptional response following sapienic acid stress in *S*. *epidermidis* and *S*. *aureus* [52]. Under these growth conditions, *S*. *aureus* upregulates urease whereas *S*. *epidermidis* upregulates ADI, the NreABC nitrogen regulation system, in addition to the nitrate and nitrite reduction pathways. The lack of NH_3_ generation via arginine catabolism in *S*. *aureus* is not unexpected as catabolism of arginine via RocF is under the control of carbon catabolite repression and CcpA [30, 31]. Our results suggest that when *S*. *aureus* is growing in the presence of glucose or another preferred carbon source, urease must utilize exogenous urea to facilitate pH homeostasis. Previous results from our laboratory demonstrated that when *S*. *aureus* grows in a defined medium lacking glucose, arginine is catabolized via RocF generating ornithine and urea [31]. Thus, we wanted to determine if urease was active under growth conditions where urea was generated via arginine catabolism and the medium was not acidic. These NMR experiments suggested that under neutral growth conditions, little ammonia from urea is detected (via detection of ^15^N-labeled glutamine). In fact, the majority of urea was detected in the culture medium as it is excreted, and is potentially used as a nitrogen storage molecule. Indeed, we found that a *ccpA* mutant grown in TSB lacking urea was able to survive weak acid stress via derepression of global amino acid catabolism. This observation suggests that when *S*. *aureus* is growing in acidic environments where peptide and amino acids are the major carbon source, arginine catabolism and urease activity is not required to facilitate pH homeostasis, which is due to rapid catabolism of amino acids and subsequent NH_3_ release.

Previous transcriptional analyses have suggested that *ureABCEFGD* is upregulated upon acid stress [10, 11, 53]. In the current study, we confirmed via β-galactosidase assays that the transcription of the *ure* genes was inhibited when the medium was buffered to a pH of 7.25 with MOPS (Fig 2A). Moreover, we found that the transcription of the urease genes was activated by CcpA and Agr, while inhibited by CodY (Fig 2B). The regulation of urease transcription by CcpA is supported by the putative catabolite-responsive element (*cre*) site identified 139 base pairs upstream of the *ureA* start codon [24]. Also, CcpA activation of *ure* transcription agrees with a previous finding that *S*. *aureus* urease, as a part of the CcpA regulon, has higher transcription as well as enzymatic activity in WT as compared to a *ΔccpA* mutant [24]. The significant decrease in *ure* gene transcription in the *Δagr* mutant that we observed is consistent with the transcriptional array data documenting that urease genes are upregulated by Agr [25]. The involvement of the urease genes in the Agr regulon strengthens the link between urease activity and virulence in *S*. *aureus*. Importantly, phagosomal acidification induces Agr activity, which is essential for *S*. *aureus* survival inside macrophages [54]. Under this circumstance, Agr may upregulate urease to counter acidic pH in coordination with enhanced virulence. CodY is also a global regulator that controls the expression of a variety of genes in gram-positive bacteria [55]. In particular, CodY senses the level of branched-chain amino acids and intracellular GTP and controls the transcription of many metabolic genes that are involved in amino acid synthesis, TCA cycle, and carbon overflow metabolism [56]. Our results agreed with previous reports that CodY represses urease gene transcription in *Bacillus subtilis* [57, 58] and *S*. *salivarius* [26]. Although urease genes are not the direct targets of CodY in *S*. *aureus* UAMS-1 [59], it is possible that CodY negatively regulates urease gene transcription through repressing Agr, since the *agrA* gene encoding the Agr response regulator is upregulated in the UAMS-1 *ΔcodY* mutant [59]. More in-depth future studies are required regarding the regulation of urease, as other transcriptional regulators such as Sae [60, 61], ClpP [62–64], and MgrA [65], are suggested to contribute to the regulatory network that fine-tunes urease activity. Lastly, it is interesting that CcpA activates *ure* transcription but represses *rocF* (arginase) transcription. These data suggest that the generation of urea by arginase is not linked to urease activity. Thus, *ure* transcription is activated when *S*. *aureus* is growing with a preferred carbon source such as glucose, which generates weak acids such as lactate or acetate. However, this also suggests that the urea utilized by urease must be exogeneous and not generated by arginase activity, which is only active when *S*. *aureus* is growing on non-preferred carbon sources such as peptides and amino acids.

To interrogate the function of urease *in vivo*, we hypothesized that the kidney is a favorable niche for *S*. *aureus* colonization and resisting the host immune response for the following reasons. First, renal blood flow is about 20% of the cardiac output [66]. Thus, *S*. *aureus* has ample opportunities to invade kidney tissue during blood filtration. Second, as urea becomes concentrated when transported through renal tubules during the production of urine, the collecting ducts in the inner medulla display the highest permeability to urea [67]; hence, not only the renal tubules but also the medullary interstitium is rich in urea, providing sufficient substrates for urease. Third, kidney medullary interstitium has a low pH (~5.5), comparing to the neutral cortical interstitium pH (~7.4) [68, 69]. In our mouse *S*. *aureus* bacteremia model, the temporal distribution of the organ bacterial burden followed what has been previously documented (S4 Fig) [40, 70, 71]. Among all the examined organs, the kidney was the only niche that developed a chronic infection over time. On days 12 and 19, mice inoculated with the *Δure* mutant had a significant decrease in CFU count in the kidneys (Fig 5), indicating a selective pressure in *S*. *aureus* to maintain urease function, similar to what has been reported in *H*. *pylori* [19]. The increased persistence of JE2 WT over *Δure* during chronic kidney infections demonstrated that urease functionality enhances the fitness of *S*. *aureus* within low pH and high urea environments such as the kidney. In order to determine whether differences in leukocyte recruitment are responsible for the changes in bacterial persistence between JE2 WT and *Δure*, individual kidneys were analyzed by flow cytometry (S5 Fig). Overall, no significant changes in the leukocyte populations were observed, indicating that urease primarily enhances bacterial persistence rather than directly altering leukocyte infiltration. The reason why we chose to evaluate the leukocyte populations on day 8 was that the kidney infections started to be cleared on approximately day 12 (Fig 5). Thus the drastic differences in the bacterial burden between JE2 WT and *Δure* could skew the immune responses on day 12. Further studies are required to determine the mechanism of how urease facilitates survival during infection. It is also possible that urease allows for persistence in the phagolysosomes upon phagocytosis by macrophages [72]. As macrophages are found in renal medulla [73], *S*. *aureus* needs to employ strategies to survive within or escape from the phagolysosomes during colonization in the kidney. Moreover, the phagolysosomes are acidic in pH, which may induce urease activity for acid resistance and survival of *S*. *aureus* [74]. Indeed, anti-inflammatory macrophages, which are prevalent during late stages of *S*. *aureus* infection, produce urea via arginase-1 [75]. For the above reasons, it would be appropriate to expand our future studies to examine the function of urease in phagolysosome survival or the escape of *S*. *aureus*, especially regarding kidney macrophages.

In summary, we identified that urease in *S*. *aureus* functions to facilitate pH homeostasis and survival under weak acid stress in the presence of urea; in non-acidic conditions, the endogenous urea derived from arginine is secreted extracellularly but not catabolized to fuel nitrogen metabolism. We found that urease is induced by weak acid stress and is within the regulation network that consists of CcpA, Agr, and CodY, interconnecting *S*. *aureus* stress response, metabolism, and virulence. We illustrated that urease provides a fitness advantage for *S*. *aureus* to persist during chronic kidney colonization of mice. These data all point to the conclusion that urease is not only a critical component of the acid stress response system of *S*. *aureus*, it is also an important factor in *S*. *aureus* pathogenesis.

## Materials and methods

### Ethics

Animal experiments were performed in ABSL2 facilities in accordance with a protocol (#11-076-08-FC) approved by the Institutional Animal Care and Use Committee (IACUC). All animals at the University of Nebraska Medical Center are maintained in compliance with the Animal Welfare Act and the Department of Health and Human Service “Guide for the Care and Use of Laboratory Animals.” Animals were anesthetized with ketamine and xylazine. Post injection of *S*. *aureus* retro-orbitally, all anesthesized mice were continuously monitored until they regained sternal recumbency and were capable of holding their heads up. The animals were monitored once/day on a daily basis following infection to ensure animal welfare. At all monitoring intervals, post-infection general appearance and body weights were recorded. Animals were euthanized by exposure to CO2 in a chamber (chamber was not pre-charged). Animals were in the CO2 filled chamber for 5 minutes after all evidence of respiration and cardiac function was absent. CO_2_ was chosen as a method of euthanasia because it has a rapid anesthetic effect and quickly results in loss of consciousness and respiratory arrest.

### Bacterial strains, plasmids and growth conditions

The *E*. *coli*, *S*. *aureus*, and *S*. *epidermidis* strains, plasmids, as well as primers used in this study are listed in S1 Table. *E*. *coli* cultures were grown in Luria-Bertani broth (LB; Difco; Becton, NJ). *S*. *aureus* and *S*. *epidermidis* were grown in tryptic soy broth (TSB; Difco; Becton, NJ) containing 14 or 45 mM glucose. CDM was prepared essentially as previously described [34], and no glucose was added. Overnight cultures grown in TSB were washed with phosphate-buffered saline (PBS) twice before inoculation to an optical density at 600 nm (OD_600_) of 0.05. Cultures were grown aerobically at 37°C in flasks with a 10:1 flask-to-volume ratio shaking at 250 rpm. When necessary, antibiotics were added to cultures as follows: ampicillin (50 μg/ml); erythromycin (10 μg/ml); tetracycline (10 μg/ml); and chloramphenicol (10 μg/ml). Bacterial growth yield was assessed by measuring the OD_600_. Culture pH was measured with a pH meter (Mettler Toledo, Columbus, OH). Bacterial viability was measured as CFU/ml by serial dilutions on TSB agar plates.

### Molecular genetic techniques

PCR amplifications were performed using Q5 High-Fidelity DNA polymerase (New England Biolabs, Beverly, MA), Midas Mix (Monserate Biotechnology Group, San Diego, CA), and oligonucleotides (S1 Table) synthesized by Sigma-Aldrich (St. Louis, MO). Restriction endonucleases and ligase from New England Biolabs (Beverly, MA) were used for DNA digestion and ligation. Purification of DNA fragments prior to subsequent cloning steps was achieved by recovery from agarose gels using a DNA Clean and Concentrator-5 Kit (Zymo Research, Orange, CA). Recombinant plasmids were purified using a Zyppy Plasmid Miniprep Kit (Zymo Research, Orange, CA). All plasmid inserts were sequenced at Eurofins Genomics (Louisville, KY) to ensure the absence of mutations.

The reporter plasmid pNF315 was constructed by amplifying the intergenic region upstream of *ureA* with primers 2833 and 2835 so that the native ribosomal binding site (RBS) was replaced with a plasmid-encoded RBS. The DNA fragment was digested and ligated into the BamHI and XhoI sites of the vector plasmid pJB185, which contains a promoterless *lacZ* [76]. pNF315 was electroporated into *S*. *aureus* RN4220 and subsequently transduced into JE2 strains using bacteriophage Φ11-mediated transduction [77].

To create the markerless JE2 *Δure* mutant, the allelic exchange plasmid pNF320 was generated by inserting the DNA sequences 1 kb upstream and 1 kb downstream of the *ureABCEFGD* operon into the temperature-sensitive *E*. *coli*-*S*. *aureus* shuttle vector plasmid pJB38 [78], using a NEBuilder HiFi DNA Assembly Cloning Kit (New England Biolabs, Beverly, MA), with primers 2980 and 2983, as well as primers 2986 and 2987. pNF320 was electroporated into *S*. *aureus* RN4220 and subsequently transduced into JE2 WT using bacteriophage Φ11-mediated transduction. Once the plasmid pNF320 was introduced into JE2, the allelic replacement to introduce the deletion mutation into the *S*. *aureus* chromosome was performed as previously described [79]. The deletion of the urease operon was confirmed phenotypically by plating on a Christensen's urea agar plate and by PCR using primers 2984 and 2985.

Chromosomally complementation of *Δure* was performed as previously described [80]. Briefly, plasmid pNF363 was constructed containing *ureABCEFGD* genes with their native promoter by amplifying an approximately 5.5 kb region from the JE2 genome using primers 2991 and 3306. The resulting DNA fragment was inserted into BamHI and PstI sites of the shuttle vector pJC1111 yielding pNF363. pJC1111 and pNF363 were subsequently transformed into RN9011 for chromosomal integration. Φ11 mediated transduction was performed to move the integrated pJC1111 and pNF363 into both JE2 WT and JE2 *ureB*::*ΦΝΣ*.

### Metabolite assays

For all metabolite assays, 1 ml bacterial culture was collected and pelleted for 2.5 min at 15,000 rpm. The supernatant was collected and stored at -80°C until use. Glucose, acetate, urea, and ammonia concentrations were determined using commercial kits (R-Biopharm AG, Darmstadt, Germany) according to the manufacturer's instructions.

### NMR sample preparation

As previously described [31, 36], five independent 50 ml cultures of *S*. *aureus* JE2 WT and the *Δure* mutant were grown to stationary phase (OD_600_ = 1.9) in CDM containing ^15^N_2_-labeled arginine (Isotec, Sigma-Aldrich, Miamisburg, OH) or ^15^N-labeled serine (Isotec, Sigma-Aldrich, Miamisburg, OH). For each culture, a total OD_600_ of 40 was collected and pelleted by centrifugation at 4000 rpm for 5 min at 4°C. 2 ml culture supernatant was collected as the media sample. Pellets were washed with 10 ml of cold sterile water twice and resuspended in 1 ml cold sterile water. The cells were lysed using a bead ruptor (OMNI International, Kennesaw, GA) and centrifuged at 15,000 rpm for 15 min at 4°C. The pellet was re-extracted with 1 ml cold sterile water. The combined cell lysate supernatant from both extractions, as well as the culture supernatant, were snap frozen in liquid nitrogen and lyophilized using a FreeZone freeze dryer.

### NMR data collection and analysis

The data collection and analysis of NMR was conducted as previously described [36]. A Bruker AVANCE IIIHD 700 MHz spectrometer equipped with a 5 mm quadruple resonance QCI-P cryoprobe (^1^H, ^13^C, ^15^N, and ^31^P), an automatic tune and match system (ATM), and a SampleJet automated sample changer system with Bruker ICON-NMR software were utilized. The 2D ^1^H−^15^N HSQC spectra collected for *S*. *aureus* cell lysates and culture media were assigned using a database of 2D 1H−15N HSQC reference spectra for known metabolites [36]. A chemical shift tolerance of 0.08 ppm for ^1^H and 0.25 ppm for ^15^N were used to match metabolites to our reference database.

### β-galactosidase assays

Beta-galactosidase assays were performed essentially as previously described [81]. Briefly, overnight cultures of JE2/pNF315 grown in TSB were inoculated in TSB containing 45 mM glucose, TSB containing 45 mM glucose buffered with 100 mM MOPS, TSB containing 45 mM glucose with 10 mM urea, and TSB containing 45 mM glucose with 10 mM urea buffered with 100 mM MOPS. At 2 h and 6 h, 2 ml and 0.5 ml of cells were collected and centrifuged (Fig 3A). Additionally, overnight cultures of JE2/pNF315, *ΔccpA*/pNF315, *Δagr*/pNF315, *ΔcodY*/pNF315 grown in TSB were inoculated to TSB containing 45 mM glucose and 10 mM urea. At 2 h, 6 h, and 10 h, 2 ml, 0.5 ml, and 0.5 ml of cells were collected and centrifuged respectively (Fig 3D). The cell pellets were resuspended in 1.2 ml Z-buffer (60 mM Na_2_HPO_4_, 40 mM NaH_2_PO_4_, 10 mM KCl, 1 mM MgSO_4_, 50 mM β-mercaptoethanol, pH 7.0) and lysed with a bead ruptor (OMNI International, Kennesaw, GA). 700 μl supernatant of the cell lysate was collected, and 140 μl of 4 mg/ml ortho-nitrophenyl-β-galactoside (ONPG) was added. The samples were incubated at 37°C until the color turned slightly yellow (under OD_420_ 1.0). 200 μl of 1 M Na_2_CO_3_ was added to stop the reaction. Protein concentrations were determined by Bradford assays using the Protein Assay Dye Solution (Bio-Rad, Hercules, California). Absorbances at 420 nm and 550 nm were measured with an Infinite 200 plate reader (Tecan, Männedorf, Switzerland).

### Amino acid analysis

Overnight cultures of JE2 WT and *ΔccpA* were inoculated to an OD_600_ of 0.05 in TSB containing 45 mM glucose. At 0 h, 3 h, 6 h, 9 h, and 12 h, 0.5 ml culture was collected and pelleted for 3 min at 15,000 rpm. Supernatant was collected and filtered through the Pierce Protein Concentrators (3,000 molecular weight cutoff; Thermo Scientific, Rockford, IL) according to the manufacturer’s instructions. Amino acid analysis was performed with a Hitachi L-8800 amino acid analyzer by the Protein Structure Core Facility, University of Nebraska Medical Center.

### Flow cytometry

The flow cytometry analyses following the growth assays were performed as previously described [7], using a BD LSRII flow cytometer (Becton and Dickinson, San Jose, California). Cells collected at 24 h and 72 h from the growth assay where JE2 WT and *ureB*::*ΦΝΣ* were cultured in TSB containing 45 mM glucose and 10 mM urea. Cell samples were washed with PBS to a final concentration of 10^7^ cells/ml and stained with 5-cyano-2,3-ditolyl tetrazolium chloride (CTC, 5 mM) and 3-(p-hydroxyphenyl) fluorescein (HPF, 15 μM). The fluorescence-activated cell sorting (FACS) was performed at a flow rate of ∼1,000 cells per second with 10,000 events per sample. Samples were excited at 488 nm, with HPF emission being detected at 530±30 nm, and CTC emission being detected at 695±40 nm. The FlowJo software was used to analyze the raw data.

For the flow cytometry analyses following the animal experiments, kidneys were collected in 1.0 mL of FACS buffer, which was composed of PBS and 2% heat-inactivated fetal bovine serum (FBS). Kidneys were homogenized with the blunt end of a 3.0 mL syringe and filtered through a 70 μm filter (BD Falcon, BD Biosciences). Filtrate was washed with PBS and collected by centrifugation (300 x g, 5 min), whereupon the filtrate was digested with Collagenase A and DNase while mixing at 37°C. The reaction was stopped after 15 minutes with heat-inactivated FBS on ice, filtered, and washed with FACS buffer, whereupon red blood cells (RBC) were lysed using the RBC Lysis Buffer (BioLegend, San Diego, CA). Single cell suspensions were washed and resuspended in FACS buffer and incubated with TruStain fcX (BioLegend, San Diego, CA) to minimize non-specific antibody binding. Samples were divided in two to analyze innate immune cell (MDSCs, neutrophils, monocytes, and macrophages) populations and lymphocyte (CD3, CD4, CD8, and γδ T cells) populations separately. Both samples were stained with Live/Dead Fixable Blue Dead Cell Stain (Invitrogen, Eugene, OR). Innate immune cells were stained with CD45-APC, Ly6G-PE, Ly6C-PerCP-Cy5.5, and F4/80-PE-Cy7, CD11b-FITC (BioLegend, San Diego, CA). Lymphocytes were stained with CD45-PE-Cy7, CD3-APC, CD4-PacBlue, CD8-FITC, and γδTCR-PE. An aliquot of pooled cells was stained with isotype-matched control antibodies to assess the degree of non-specific staining per treatment group [82]. For individual samples, 10,000–100,000 events were analyzed using BD FACSDiva software with cell populations expressed as percentage of total viable CD45^+^ leukocytes.

### Animal experiments

Seven-week-old male and female C57BL/6 mice (Charles River Laboratories, Wilmington, MA) were used in all animal experiments. Overnight cultures of *S*. *aureus* JE2 WT and *Δure* in TSB were washed with PBS twice and suspended in PBS to yield an OD_600_ of 10. The cultures were further diluted 1:50 with PBS, prior to the retro-orbital injection of 50 μl (10^6^ CFU) final bacterial suspension. The inocula were verified by serial dilution plating and colony enumeration on TSB agar plates. Mice were anesthetized by intraperitoneal injection of ketamine/xylazine (60 mg/kg and 3 mg/kg, respectively). Mice were euthanized for the quantification of bacterial burden (expressed as Log [(CFU/g of tissue) +1]), by serial dilution plating and colony enumeration of homogenized organs.

### Statistical methods

For all studies, statistical analysis was performed using GraphPad Prism 5.0 software (La Jolla, CA). P-values < 0.05 were considered significant. For comparisons of two groups, Mann-Whitney test was used. One-way analysis of variance (ANOVA) was performed to compare three or more groups. Two-way repeated measures ANOVA was performed to compare differences between groups with two independent variables.

## Supporting information

S1 Fig Complementation of JE2 *ureB*::*ΦΝΣ* mutant. (**A**) Cell viabilities (CFU/ml) and (**B**) pH of JE2, JE2 SaPI1 *attC*::pJC1111 (vector control), JE2 SaPI1 *attC*::pNF363 (complement), JE2 *ureB*::*ΦΝΣ*, JE2 *ureB*::*ΦΝΣ* SaPI1 *attC*::pJC1111, JE2 *ureB*::*ΦΝΣ* SaPI1 *attC*::pNF363 were monitored every 24 h over 5 days in TSB containing 45 mM glucose and 10 mM urea (n = 3/strain, mean ± SEM). Starter cultures were grown overnight in TSB containing 14mM glucose and 10mM urea. Statistical significance was assessed using two-way repeated measures ANOVA followed by Bonferroni post-test compared to JE2 at each timepoint; *** *P <* 0.001.
(TIF)

S2 Fig Urease rescues cellular respiration and prevents reactive oxygen species production under weak acid stress in the presence of exogenous urea. (**A**)-(**D**) JE2 WT and JE2 *ureB*::*ΦΝΣ* were cultured in TSB containing 45 mM glucose with and without 10 mM urea. Flow cytometry density plots of cells collected at 24 h and 72 h, and double stained with HPF and CTC. Data shown are a representative of 3 biological replicates. (**A**) 24 h, CTC staining. (**B**) 24 h, HPF staining. (**C**) 72 h, CTC staining. (**D**) 72 h, HPF staining. CTC accumulates in the actively respiring cells, and HPF is indicative of ROS production.
(TIF)

S3 Fig Different than *S*. *epidermidis*, *S*. *aureus* arginine deiminase is less active than urease in rescuing cell death under weak acid stress. (**A**) and (**B**) Five-day growth assay of *S*. *aureus* JE2 WT and JE2 *arcA1*::*kan/arcA2*:: *ΦΝΣ* in TSB containing 45 mM glucose with and without 5 mM arginine. Every 24 h, (**A**) cell viability (CFU/ml) and (**B**) extracellular pH were monitored (n = 3, mean ± SEM). (**C**) and (**D**) Five-day growth assay of *S*. *epidermidis* 1457 WT in TSB containing 35 mM glucose with and without 5 mM arginine. Every 24 h, (**C**) cell viability (CFU/ml) and (**D**) extracellular pH were monitored (n = 3, mean ± SEM). Statistical significance was assessed using two-way repeated measures ANOVA followed by Bonferroni post-test; * *P <* 0.05, *** *P <* 0.001. (**E**) and (**F**) Five-day growth assay of *S*. *epidermidis* 1457 WT in TSB containing 35 mM glucose with and without 5 mM urea. Every 24 h, (**E**) cell viability (CFU/ml) and (**F**) extracellular pH were monitored (n = 3, mean ± SEM). Statistical significance was assessed using two-way repeated measures ANOVA followed by Bonferroni post-test; * *P <* 0.05.
(TIF)

S4 Fig *S*. *aureus* JE2 WT persists in murine kidneys over time. (**A)-(D)** *S*. *aureus* murine bacteremia model, male and female C57BL/6 mice were infected with JE2 WT. On day 2 (number of mice: n = 8), day 5 (number of mice: n = 9), day 8 (number of mice: n = 6), day 12 (number of mice: n = 8), and day 19 (number of mice: n = 7) post-inoculation, heart (**A**), liver (**B**), spleen (**C**), and kidneys (**D**) were harvested. Bacterial burdens were calculated as Log_10_ (CFU/g of tissue +1) and plotted with medians.
(TIF)

S5 Fig *S*. *aureus* urease does not influence infiltrating leukocyte populations during renal infection. Infiltrating leukocyte populations from day 8 kidneys isolated from animals infected with *S*. *aureus* JE2 WT or JE2 *Δure* were evaluated by flow cytometry. Leukocyte populations were reported as a percentage of total CD45^+^ leukocytes (mean ± SEM). (**A**) MDSCs (Ly6G^high^Ly6C^+^CD11b^high^F4/80^-^) (**B**) Neutrophils (Ly6G^high^Ly6C^+^CD11b^low^F4/80^-^) (**C**) Monocytes (Ly6G^-^Ly6C^+^CD11b^+^F4/80^-^) (**D**) Macrophages (Ly6G^-^Ly6C^-^CD11b^+^F4/80^+^) (**E**) T cells (CD3^+^) (**F**) CD4^+^ T cells (CD3^+^γδTCR^-^CD4^+^CD8^-^) (**G**) CD8^+^ T cells (CD3^+^γδTCR^-^CD4^-^CD8^+^) (**H**) γδ T cells (CD3^+^γδTCR^+^CD4^-^CD8^-^). Statistical significance was assessed using the Mann- Whitney test; ns, not significant.
(TIF)

S1 Table Strains, plasmids and primers. (DOCX)
