# Supplementary figures and images for: Urease is an essential component of the acid response network of Staphylococcus aureus and is required for a persistent murine kidney infection
Source: PLoS Pathog. 2019 Jan 4;15(1):e1007538. doi: 10.1371/journal.ppat.1007538 (PMC6343930; doi:10.1371/journal.ppat.1007538)

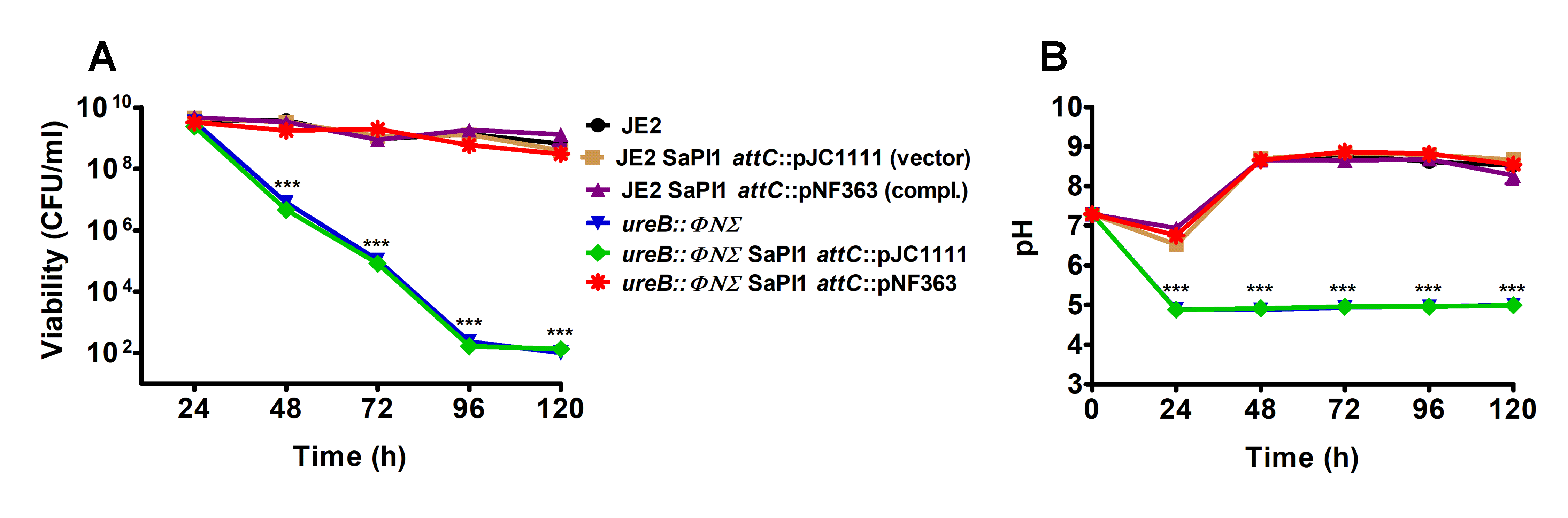

Supplement: S1 Fig — (A) Cell viabilities (CFU/ml) and (B) pH of JE2, JE2 SaPI1 attC::pJC1111 (vector control), JE2 SaPI1 attC::pNF363 (complement), JE2 ureB::ΦΝΣ, JE2 ureB::ΦΝΣ SaPI1 attC::pJC1111, JE2 ureB::ΦΝΣ SaPI1 attC::pNF363 were monitored every 24 h over 5 days in TSB containing 45 mM glucose and 10 mM urea (n = 3/strain, mean ± SEM). Starter cultures were grown overnight in TSB containing 14mM glucose and 10mM urea. Statistical significance was assessed using two-way repeated measures ANOVA followed by Bonferroni post-test compared to JE2 at each timepoint; *** P < 0.001. (TIF) [file ppat.1007538.s001.tif]

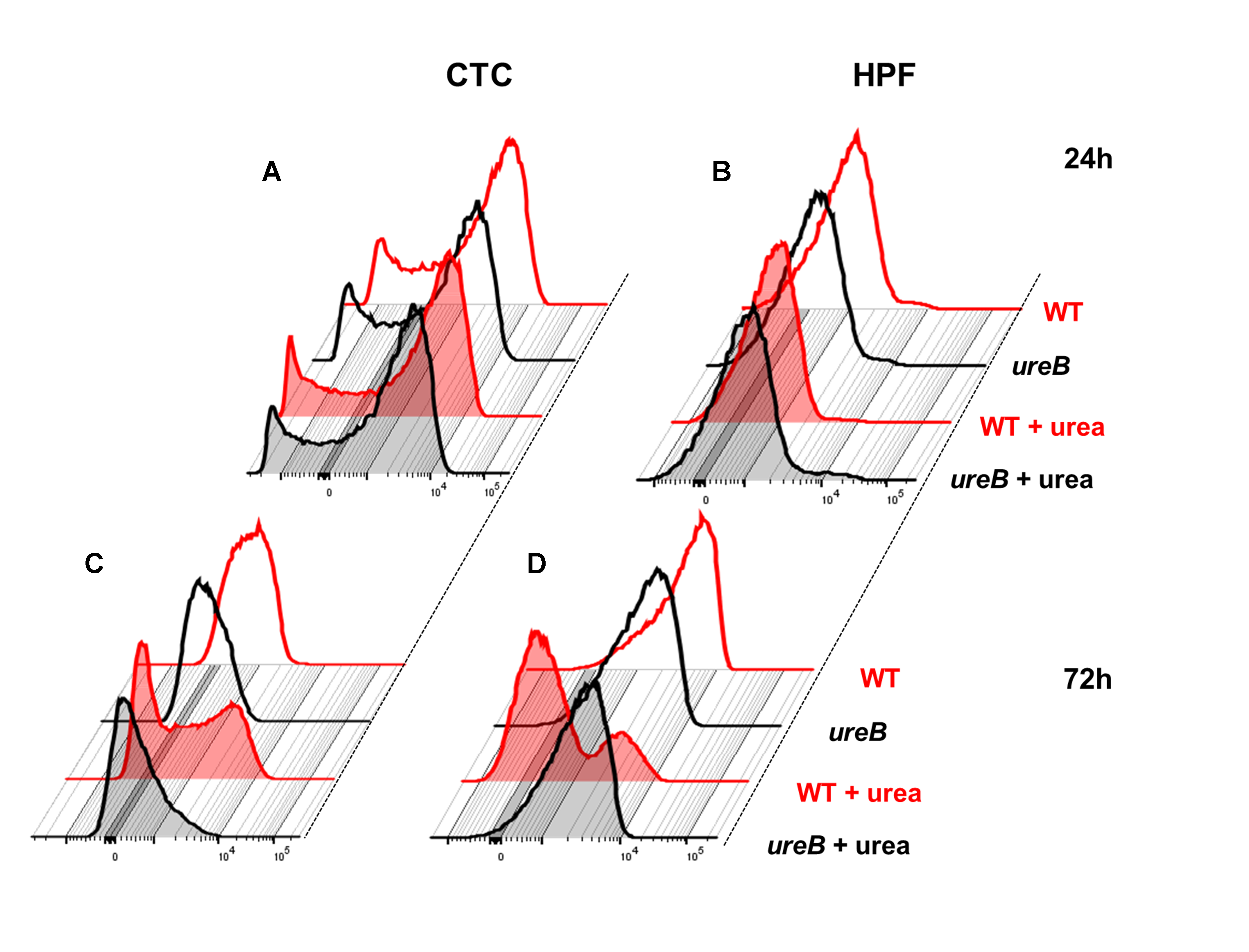

Supplement: S2 Fig — (A)-(D) JE2 WT and JE2 ureB::ΦΝΣ were cultured in TSB containing 45 mM glucose with and without 10 mM urea. Flow cytometry density plots of cells collected at 24 h and 72 h, and double stained with HPF and CTC. Data shown are a representative of 3 biological replicates. (A) 24 h, CTC staining. (B) 24 h, HPF staining. (C) 72 h, CTC staining. (D) 72 h, HPF staining. CTC accumulates in the actively respiring cells, and HPF is indicative of ROS production. (TIF) [file ppat.1007538.s002.tif]

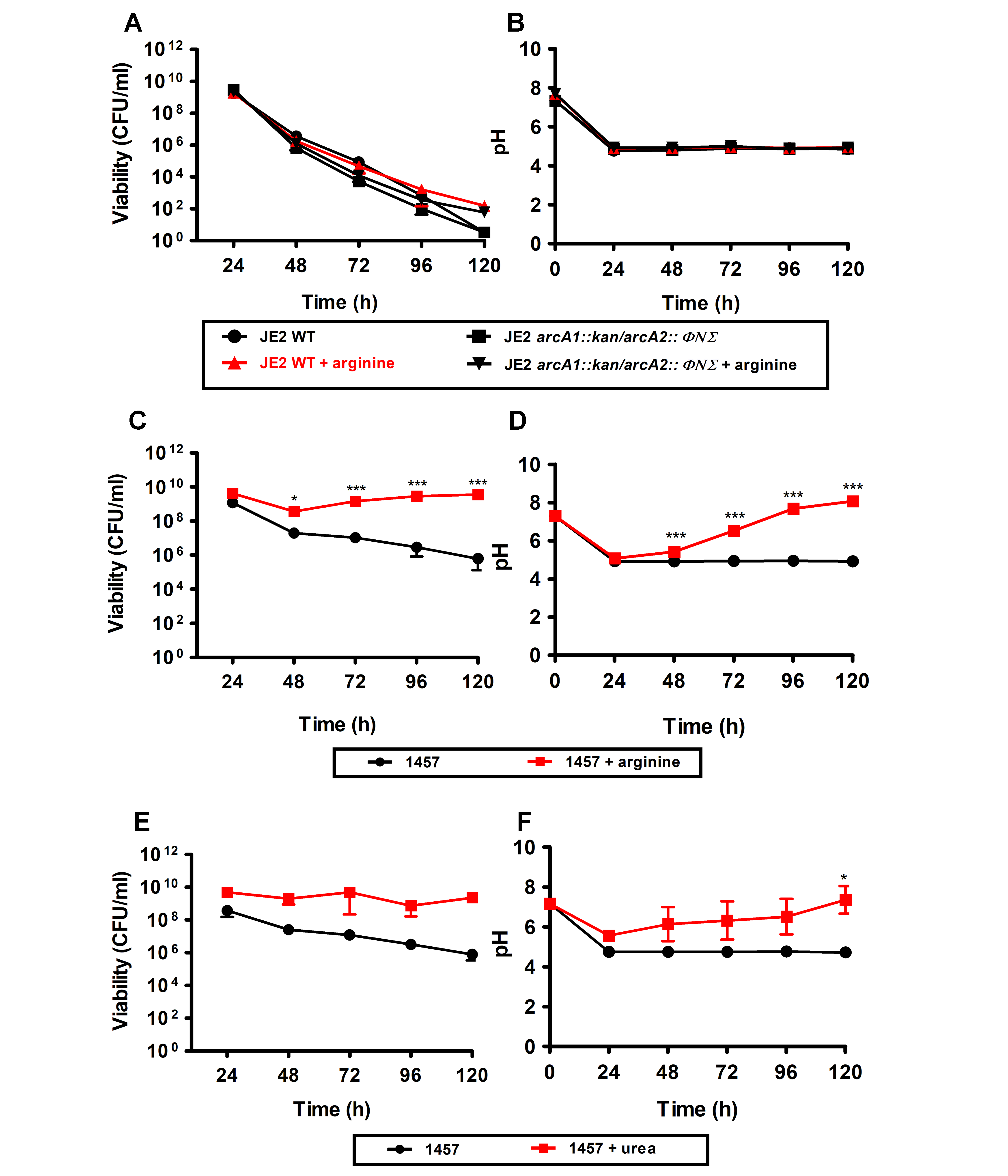

Supplement: S3 Fig — (A) and (B) Five-day growth assay of S. aureus JE2 WT and JE2 arcA1::kan/arcA2:: ΦΝΣ in TSB containing 45 mM glucose with and without 5 mM arginine. Every 24 h, (A) cell viability (CFU/ml) and (B) extracellular pH were monitored (n = 3, mean ± SEM). (C) and (D) Five-day growth assay of S. epidermidis 1457 WT in TSB containing 35 mM glucose with and without 5 mM arginine. Every 24 h, (C) cell viability (CFU/ml) and (D) extracellular pH were monitored (n = 3, mean ± SEM). Statistical significance was assessed using two-way repeated measures ANOVA followed by Bonferroni post-test; * P < 0.05, *** P < 0.001. (E) and (F) Five-day growth assay of S. epidermidis 1457 WT in TSB containing 35 mM glucose with and without 5 mM urea. Every 24 h, (E) cell viability (CFU/ml) and (F) extracellular pH were monitored (n = 3, mean ± SEM). Statistical significance was assessed using two-way repeated measures ANOVA followed by Bonferroni post-test; * P < 0.05. (TIF) [file ppat.1007538.s003.tif]

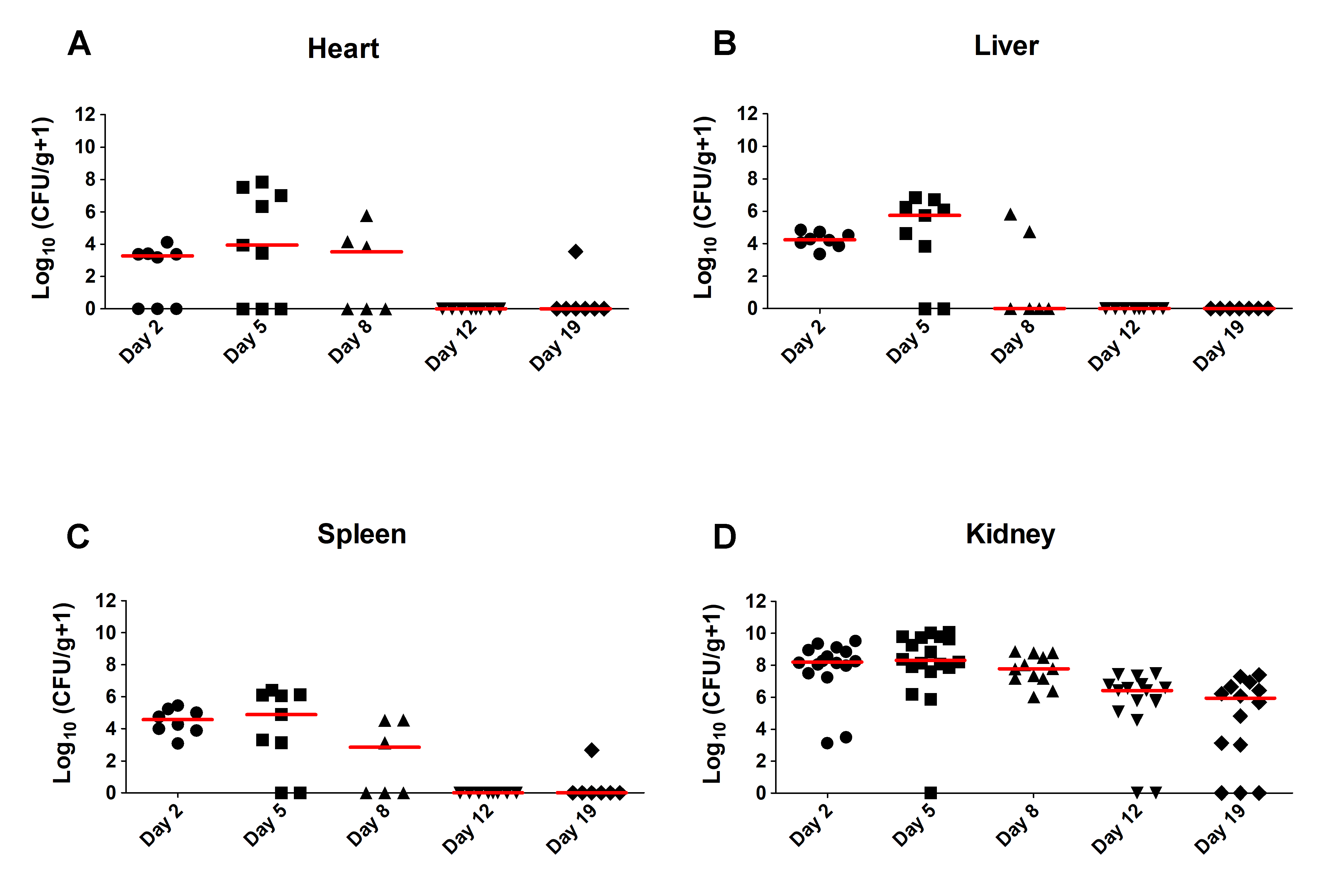

Supplement: S4 Fig — (A)-(D) S. aureus murine bacteremia model, male and female C57BL/6 mice were infected with JE2 WT. On day 2 (number of mice: n = 8), day 5 (number of mice: n = 9), day 8 (number of mice: n = 6), day 12 (number of mice: n = 8), and day 19 (number of mice: n = 7) post-inoculation, heart (A), liver (B), spleen (C), and kidneys (D) were harvested. Bacterial burdens were calculated as Log10 (CFU/g of tissue +1) and plotted with medians. (TIF) [file ppat.1007538.s004.tif]

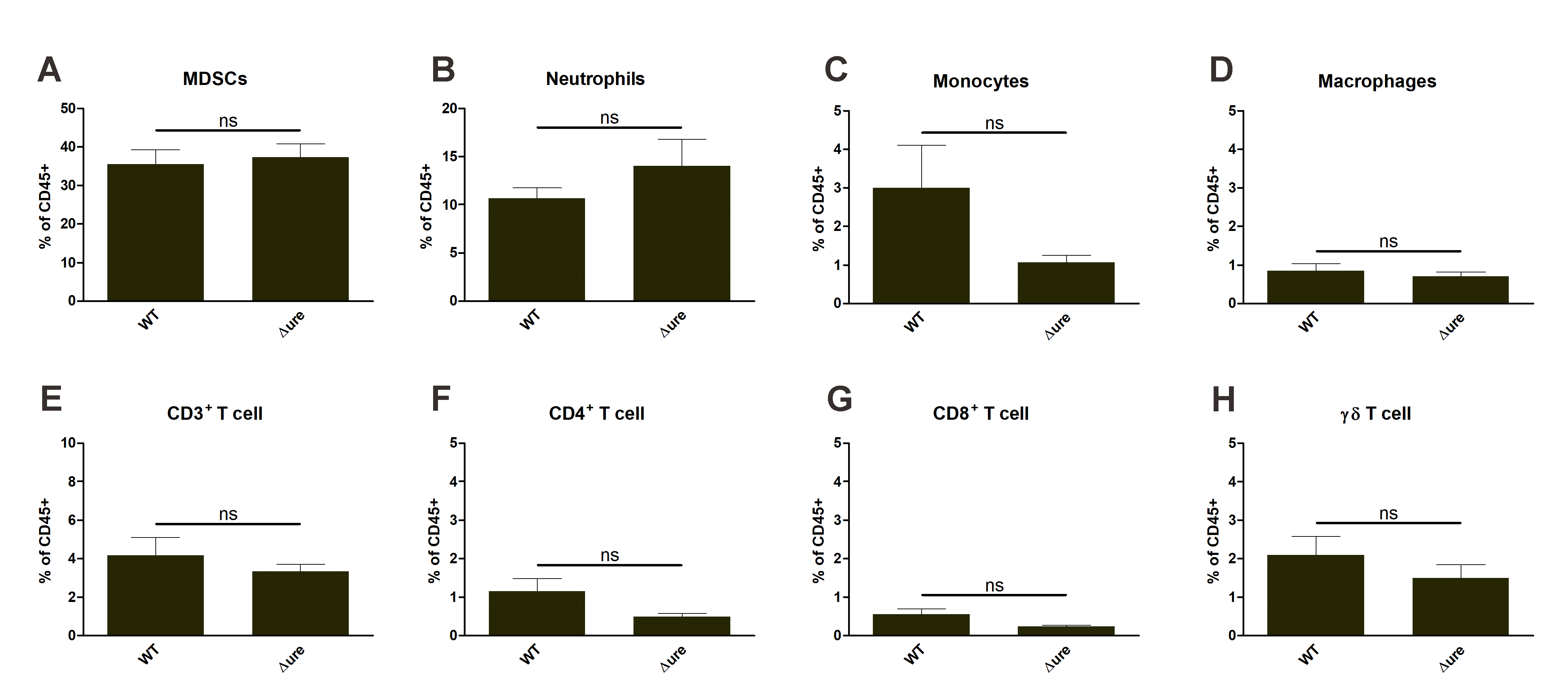

Supplement: S5 Fig — Infiltrating leukocyte populations from day 8 kidneys isolated from animals infected with S. aureus JE2 WT or JE2 Δure were evaluated by flow cytometry. Leukocyte populations were reported as a percentage of total CD45+ leukocytes (mean ± SEM). (A) MDSCs (Ly6GhighLy6C+CD11bhighF4/80-) (B) Neutrophils (Ly6GhighLy6C+CD11blowF4/80-) (C) Monocytes (Ly6G-Ly6C+CD11b+F4/80-) (D) Macrophages (Ly6G-Ly6C-CD11b+F4/80+) (E) T cells (CD3+) (F) CD4+ T cells (CD3+γδTCR-CD4+CD8-) (G) CD8+ T cells (CD3+γδTCR-CD4-CD8+) (H) γδ T cells (CD3+γδTCR+CD4-CD8-). Statistical significance was assessed using the Mann- Whitney test; ns, not significant. (TIF) [file ppat.1007538.s005.tif]
