# Supplementary material for: Urease is an essential component of the acid response network of Staphylococcus aureus and is required for a persistent murine kidney infection
Source: PLoS Pathog. 2019 Jan 4;15(1):e1007538. doi: 10.1371/journal.ppat.1007538 (PMC6343930; doi:10.1371/journal.ppat.1007538)
Supplement: S1 Table — (DOCX) [file ppat.1007538.s006.docx]

**Table S1**

**Strains, plasmids and primers**

| **Strains** | **Description** | **Source** |
| --- | --- | --- |
| ***Escherichia coli*** |  |  |
| E10B | General plasmid maintenance strain | Stratagene |
| DH5α | Cloning strain | Gibco |
| ***Staphylococcus aureus*** |  |  |
| RN4220 | Restriction deficient strain commonly used as a transformation intermediate | [1] |
| RN9011 | RN4220/pRN7023 (SaPI1 integrase, *cat194*) Cm^r^ | [2] |
| JE2 | Wildtype; CA-MRSA USA300 LAC cured of all 3 native plasmids; Erm^s^, Cm^s^, Tet^s^ | [3] |
| JE2 *ureB::ΦΝΣ* | *bursa aurealis ureB* mutation in JE2; Erm^r^ | [4] |
| JE2 *Δure* | Deletion mutant of *ureABCEFGD* in JE2 | This study |
| JE2 *ΔccpA**::tetL* | Allelic replacement mutation in *ccpA* in JE2; Tet^r^ | [3] |
| JE2 *Δagr::tetM* | Allelic replacement mutation in agr in JE2; transduced from AH1292; Tet^r^ | This study |
| JE2 *ΔcodY::ermB* | Allelic replacement mutation in *codY* in JE2, transduced from *ΔcodY::ermB* in LAC; Erm^r^ | This study |
| JE2 *arcA1::kan/arcA2::ΦΝΣ* | *bursa aurealis arcA1 and arcA2* mutations, the erm cassette exchanged with kan in *arcA1*; Kan^r^, Erm^r^ | [5] |
| JE2 *ΔccpA::tetL/gudB::ΦΝΣ* | *bursa aurealis gudB* mutation in JE2 *ccpA::tetL*; Tet^r^, Erm^r^ | This study |
| JE2 *ΔccpA::tetL/Δure* | *ureABCEFGD* deletion mutant in JE2 *ΔccpA::tetL*; Tet^r^ | This study |
| JE2 *ΔccpA::tetL/rocF::ΦΝΣ* | *bursa aurealis rocF* mutation in JE2 *ΔccpA::tetL*; Tet^r^, Erm^r^ | [5] |
| JE2 *ΔccpA::tetL/putA::ΦΝΣ* | *bursa aurealis putA* mutation in JE2 *ΔccpA::tetL*; Tet^r^, Erm^r^ | [3] |
| JE2 *ΔccpA::tetL/arcA1::kan/arcA2::ΦΝΣ* | *ccpA*, *arcA1*, *arcA2* triple mutant in JE2; transduced from JE2 *arcA1::kan/arcA2::ΦΝΣ* into JE2 *ΔccpA::tetL*; Tet^r^, Kan^r^, Erm^r^ | This study |
| JE2/pNF315 | JE2 containing pNF315; Cm^r^ | This study |
| JE2 *ΔccpA::tetL*/pNF315 | JE2 *ΔccpA::tetL* containing pNF315; Tet^r^, Cm^r^ | This study |
| JE2 *Δagr::tetM*/pNF315 | JE2 *Δagr::tetM* containing pNF315; Tet^r^, Cm^r^ | This study |
| JE2 *ΔcodY::ermB*/pNF315 | JE2 *ΔcodY::ermB* containing pNF315; Erm^r^, Cm^r^ | This study |
| JE2 SaPI1 *attC*::pJC1111 | JE2 with pJC1111 integrated to the SaPI1 *attC* site | This study |
| JE2 SaPI1 *attC*::pNF363 | JE2 with pNF363 integrated to the SaPI1 *attC* site | This study |
| JE2 *ureB::ΦΝΣ* SaPI1 *attC*::pJC1111 | JE2 *ureB::ΦΝΣ* with pJC1111 integrated to the SaPI1 *attC* site | This study |
| JE2 *ureB::ΦΝΣ* SaPI1 *attC*::pNF363 | JE2 *ureB::ΦΝΣ* with pNF363 integrated to the SaPI1 *attC* site | This study |
| ***Staphylococcus epidermidis*** |  |  |
| 1457 | Wild-type strain used in study | [6] |
|  |  |  |
| **Plasmids** |  |  |
| pJB38 | Temperature-sensitive allelic exchange plasmid; Amp^r^,Cm^r^ | [7] |
| pJB185 | Promoterless codon-optimized *lacZ* containing plasmid; Amp^r^, Cm^r^ | [8] |
| pNF315 | JE2 *ureABCEFGD* promoter::*lacZ* reporter plasmid; Amp^r^, Cm^r^ | This study |
| pNF320 | JE2 *Δure* allelic exchange plasmid; Amp^r^, Cm^r^ | This study |
| pJC1111 | SaPI1 *attS* suicide vector, Cad^r^, | [10] |
| pNF363 | *ureABCEFGD* genes with the native promoter cloned into pJC1111 | This Study |

| **Primers** |  |  |
| --- | --- | --- |
| **Name** | **Sequence (5' to 3')** | **Target** |
| 2833 | GCGGATCCCCTATTAAGATAAATAATCCTGTCC | Forward primer to amplify the intergenic region containing the *ure* promoter |
| 2835 | CGCTCGAGCAAAATTTATATTAATCCTAATTGTTGTG | Reverse primer to amplify the intergenic region containing the *ure* promoter in which the native ribosomal biding site (RBS) is replaced with a plasmid coded RBS |
| 2980 | CCTTTCGTCTTCAAGAATTCTAATCGCTGTTTTAAATGTATATCC | Forward primer to amplify the fragment upstream of *ure* for making pNF320 |
| 2981 | TCTTTTTATTTTACAAATTAAAACCCCCAATTTC | Reverse primer to amplify the fragment upstream of *ure* for making pNF320 |
| 2982 | GTTTTAATTTGTAAAATAAAAAGACTAGTGTACCTTG | Forward primer to amplify the fragment downstream of *ure* for making pNF320 |
| 2983 | TTGCATGCCTGCAGGTCGACTTGTTGAATATGTAAAACAACATATG | Reverse primer to amplify the fragment downstream of *ure* for making pNF320 |
| 2984 | GCCGCTAATCCAATTGTCCAA | Forward primer to confirm *ure* deletion |
| 2985 | GAGATTGCTAAGTGCTCAGAG | Reverse primer to confirm *ure* deletion |
| 2986 | GTCGACCTGCAGGCATGC | Forward primer to amplify pJB38 for making pNF320 |
| 2987 | GAATTCTTGAAGACGAAAGGGCC | Reverse primer to amplify pJB38 for making pNF320 |
| 3306 | GATCCGTCAGCCAATTGTCCAATCGGCTAC | Forward primer to amplify the *ure* operon for complementation |
| 2991 | ATGCGGATCCCACCTAAACGAATGGAATCTCC | Reverse primer to amplify the *ure* operon for complementation |

Restriction enzyme recognition sites are underlined. GGATCC, BamHI; CTCGAG, XhoI; CTGCAG, PstI

**References**

1. Kreiswirth BN, Lofdahl S, Betley MJ, O'Reilly M, Schlievert PM, Bergdoll MS, et al. The toxic shock syndrome exotoxin structural gene is not detectably transmitted by a prophage. Nature. 1983;305(5936):709-12. Epub 1983/10/20. PubMed PMID: 6226876.

2. Ruzin A, Lindsay J, Novick RP. Molecular genetics of SaPI1--a mobile pathogenicity island in *Staphylococcus aureus*. Molecular microbiology. 2001;41(2):365-77. PubMed PMID: 11489124.

3. Nuxoll AS, Halouska SM, Sadykov MR, Hanke ML, Bayles KW, Kielian T, et al. CcpA Regulates Arginine Biosynthesis in *Staphylococcus aureus* through Repression of Proline Catabolism. PLOS Pathogens. 2012;8(11):e1003033. doi: 10.1371/journal.ppat.1003033.

4. Fey PD, Endres JL, Yajjala VK, Widhelm TJ, Boissy RJ, Bose JL, et al. A genetic resource for rapid and comprehensive phenotype screening of nonessential *Staphylococcus aureus* genes. mBio. 2013;4(1):e00537-12. doi: 10.1128/mBio.00166-13. PMID: 23549918; OID: NLM: PMC3573662; epublish

10.1128/mBio.00537-12 [doi].

5. Halsey CR, Lei S, Wax JK, Lehman MK, Nuxoll AS, Steinke L, et al. Amino Acid Catabolism in *Staphylococcus aureus* and the Function of Carbon Catabolite Repression. mBio. 2017;8(1):10.1128/mBio.01434-16. doi: e01434-16 [pii].

6. Mack D, Siemssen N, Laufs R. Parallel induction by glucose of adherence and a polysaccharide antigen specific for plastic-adherent *Staphylococcus epidermidis*: evidence for functional relation to intercellular adhesion. Infect Immun. 1992;60(5):2048-57. Epub 1992/05/01. PubMed PMID: 1314224; PubMed Central PMCID: PMCPMC257114.

7. Bose JL, Fey PD, Bayles KW. Genetic tools to enhance the study of gene function and regulation in *Staphylococcus aureus*. Applied and Environmental Microbiology. 2013;79(7):2218-24. doi: 10.1128/AEM.00136-13 [doi].

8. Krute CN, Rice KC, Bose JL. VfrB Is a Key Activator of the *Staphylococcus aureus* SaeRS Two-Component System. J Bacteriol. 2017;199(5). Epub 2016/12/30. doi: 10.1128/jb.00828-16. PubMed PMID: 28031278; PubMed Central PMCID: PMCPMC5309915.

9. Lee CY, Buranen SL, Ye ZH. Construction of single-copy integration vectors for *Staphylococcus aureus*. Gene. 1991;103(1):101-5. Epub 1991/07/15. PubMed PMID: 1652539.

10. Geisinger E, George EA, Chen J, Muir TW, Novick RP. Identification of ligand specificity determinants in AgrC, the *Staphylococcus aureus* quorum-sensing receptor. J Biol Chem. 2008;283(14):8930-8. doi: 10.1074/jbc.M710227200. PubMed PMID: 18222919; PubMed Central PMCID: PMCPMC2276371.
